# Shared genetic etiology and causality between body fat percentage and cardiovascular diseases: a large-scale genome-wide cross-trait analysis

**DOI:** 10.1186/s12916-021-01972-z

**Published:** 2021-04-29

**Authors:** Zhenhuang Zhuang, Minhao Yao, Jason Y. Y. Wong, Zhonghua Liu, Tao Huang

**Affiliations:** 1 Department of Epidemiology & Biostatistics, School of Public Health, Peking University, China. 38 Xueyuan Road, Beijing, 100191 China; 2 Department of Statistics and Actuarial Science, The University of Hong Kong, Hong Kong, China; 3 Division of Cancer Epidemiology and Genetics, National Cancer Institute, National Institutes of Health, Rockville, MD USA; 4 Center for Intelligent Public Health, Academy for Artificial Intelligence, Peking University, Beijing, 100191 China; 5 Key Laboratory of Molecular Cardiovascular Sciences (Peking University), Ministry of Education, Beijing, 100191 China

**Keywords:** Body fat percentage, Cardiovascular diseases, Shared genetics, Genetic correlation, Mendelian randomization

## Abstract

**Background:**

Accumulating evidences have suggested that high body fat percentage (BF%) often occurs in parallel with cardiovascular diseases (CVDs), implying a common etiology between them. However, the shared genetic etiology underlying BF% and CVDs remains unclear.

**Methods:**

Using large-scale genome-wide association study (GWAS) data, we investigated shared genetics between BF% (*N* = 100,716) and 10 CVD-related traits (*n* = 6968-977,323) with linkage disequilibrium score regression, multi-trait analysis of GWAS, and transcriptome-wide association analysis, and evaluated causal associations using Mendelian randomization.

**Results:**

We found strong positive genetic correlations between BF% and heart failure (HF) (Rg = 0.47, *P* = 1.27 × 10^− 22^) and coronary artery disease (CAD) (Rg = 0.22, *P* = 3.26 × 10^− 07^). We identified 5 loci and 32 gene-tissue pairs shared between BF% and HF, as well as 16 loci and 28 gene-tissue pairs shared between BF% and CAD. The loci were enriched in blood vessels and brain tissues, while the gene-tissue pairs were enriched in the nervous, cardiovascular, and exo-/endocrine system. In addition, we observed that BF% was causally related with a higher risk of HF (odds ratio 1.63 per 1-SD increase in BF%, *P* = 4.16 × 10–04) using a MR approach.

**Conclusions:**

Our findings suggest that BF% and CVDs have shared genetic etiology and targeted reduction of BF% may improve cardiovascular outcomes. This work advances our understanding of the genetic basis underlying co-morbid obesity and CVDs and opens up a new way for early prevention of CVDs.

## Background

Body fat percentage (BF%) is a proxy for adiposity that is genetically regulated through a leptin or melanocortin pathway in the central nervous system (CNS) [1]. Recent studies found that high BF%, especially with excess visceral adipose tissue, is associated with increased risk of cardiovascular diseases (CVDs) [2, 3], independent of body mass index (BMI) [4, 5]. BF% is biologically important and distinct from other proxies of adiposity such as BMI and waist-to-hip ratio for its capacity to differentiate between fat-free mass (i.e., lean mass, bone mass and fluid mass) and fat mass [6, 7]. One hypothesis to account for the link between high BF% and CVDs is shared genetic etiology. BF% and CVDs may share common genetic variants that influence metabolism or response to environmental risk factors [8]. While the genetic basis for BF% and CVDs is poorly understood, large-scale genome-wide association studies (GWAS) may provide novel insight into specific biological processes underlying their comorbidity.

Genetic correlation analysis estimates the correlation of genetic effects between two clinically related traits and highlights the shared etiologies behind such an association. With methodological advances in molecular genetics and the increased number of available GWAS results, it is now feasible for us to investigate genome-wide genetic correlation and identify significant expression-trait associations for complex traits by using genomics resources (i.e., summary-level statistics from large GWAS [8–14] and the Genotype-Tissue Expression (GTEx) project [15]) and state-of-the-art statistical analysis methods (i.e., linkage disequilibrium score regression (LDSC) [16, 17], multi-trait analysis of GWAS (MTAG) [18], and transcriptome-wide association studies (TWAS) [19]). Furthermore, previous twin and family studies have shown that BF% and CVDs are heritable traits, with heritability estimates ranging from 25 to 40% [10, 20, 21]. Large-scale GWAS have enabled detection of more than 20 susceptibility loci for BF% [8]. In particular, the identified BF%-related loci near *FTO* predicts long-term CVD risk (OR = 1.895), suggesting that BF% and CVDs might share genetic architecture [22]. Previous post-GWAS analyses conducted in a large European population found that genetically predicted BMI was strongly associated with several CVD outcomes, including heart failure (HF) and coronary artery disease (CAD) [23]. Further, there was suggestive evidence of associations between genetically predicted fat mass index and some CVDs [23]. However, these studies are limited because BMI is an imperfect measure of adiposity which does not directly measure body fat. To our knowledge, no large-scale genome-wide study has systematically reported the shared genetic loci between excess adiposity and CVDs, which is not fully accounted for through BMI evaluation.

Although observational studies have reported associations between BF% and adverse cardiovascular outcomes [24–26], some of the findings have been inconsistent, which may be due to biases such as unmeasured confounding. Mendelian randomization (MR) is a form of instrumental variable analysis that can be used to estimate the causal association under certain assumptions [27, 28], even in the presence of unmeasured confounders. HF is a catabolic state that can lead to weight reduction [29], which indicates that the association between body weight and HF could be bidirectional. Given that inherited genetic variants are unlikely to be influenced by reverse causation or environmental confounders after accounting for population stratification, investigating the directions of these associations is crucially important.

Using genome-wide association study (GWAS) summary-level data from several international consortia (*n* = 6968-977,323), we investigated the genetic correlation and causality between BF% and 10 CVD-related traits with the overarching goal of characterizing the specific shared genetic loci and biological pathways. Further, we conducted a large-scale, genome-wide cross-trait analysis to explore novel genetic components among these diseases. The biological effects reflected by shared loci may play important roles in the co-occurrence of high BF% and CVDs.

## Methods

### Study design, data summary and quality control

**Fig. 1:**
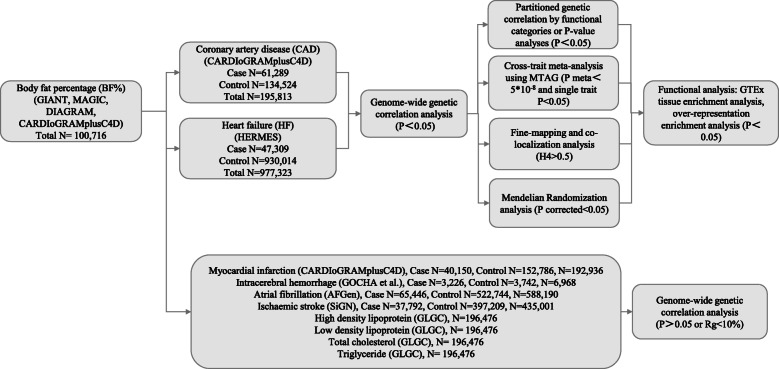
Overall study design. Multiple GWAS data sources were first retrieved. We first conducted genome-wide genetic correlation between BF% and 10 cardiovascular disease (CVD)-related traits. For CVD traits that were shown significant genetic correlation with BF%, we conducted further post-GWAS analyses to investigate genetic overlap between them (variant/region/functional levels, and causal inference). GWAS: genome-wide association study; GIANT: Genetic Investigation of ANthropometric Traits; MAGIC: Meta-Analyses of Glucose and Insulin-related traits Consortium; DIAGRAM: DIAbetes Genetics Replication And Meta-analysis; CARDIoGRAMplusC4D: the Coronary ARtery DIsease Genome wide Replication and Meta-analysis (CARDIoGRAM) plus the Coronary Artery Disease (C4D) Genetics (CARDIoGRAMplusC4D) consortium; HERMES: Heart Failure Molecular Epidemiology for Therapeutic Targets; GOCHA: Genetics of Cerebral Hemorrhage with Anticoagulation; SiGN: the NINDS Stroke Genetics Network; ISGC: International Stroke Genetics Consortium; GLGC: The Global Lipids Genetics; MTAG, multi-trait analysis of genome-wide association studies

The overall study design is shown in Fig. 1. We retrieved summary statistics from the Genetic Investigation of ANthropometric Traits (GIANT) consortium for BF% (*n* = 100,716) [8]; the Heart Failure Molecular Epidemiology for Therapeutic Targets (HERMES) for HF (47,309 cases and 930,014 controls) [9]; the Coronary ARtery Disease Genome wide Replication and Meta-analysis (CARDIoGRAM) plus the Coronary Artery Disease (C4D) Genetics (CARDIoGRAMplusC4D) consortium for CAD (60,801 cases and 123,504 controls) [10]; and myocardial infarction (MI) (43,676 cases and 128,197 controls) [10], respectively; from the NINDS Stroke Genetics Network (SiGN) and International Stroke Genetics Consortium (ISGC) for ischemic stroke (IS) (37,792 cases and 397,209 controls) [11]; from the AFGen Consortium for atrial fibrillation (AF) (65,446 cases and 522,744 controls) [12]; from the Genetics of Cerebral Hemorrhage with Anticoagulation (GOCHA) study and the Genetic and Environmental Risk Factors for Hemorrhagic Stroke (GERFHS) studies for intracerebral hemorrhage (ICH) (3226 cases and 3742 controls) [13]; and from the Global Lipids Genetics Consortium (GLGC) consortium (*n* = 188,578) for lipids such as high-density lipoprotein (HDL) [14], low-density lipoprotein (LDL) [14], total cholesterol (TC) [14], and triglycerides (TG) [14]. Each study has done the study-specific quality control to ensure the criteria of BF% measurement and CVDs similar among the different studies. Baseline characteristics for each study are also provided in previous studies. For example, BF% in each cohort was measured either with bioimpedance analysis or dual energy X-ray absorptiometry as described in detail before [8]. In addition, CAD status was defined by an inclusive CAD diagnosis, including MI, chronic stable angina, acute coronary syndrome, or coronary stenosis > 50% [10]. HF cases included participants with a clinical diagnosis of HF of any etiology with no inclusion criteria based on LV ejection fraction [9]. Details of each of the GWAS studies are present in Table S1.

The ethical approval and quality control procedures of each consortium have been described in previous studies [8–14]. Informed consent was obtained from all participants of contributing studies. In addition, we restricted the chromosome region to autosomal chromosomes and excluded single-nucleotide polymorphisms (SNPs) in MHC region (chr6:25 Mb–34 Mb).

### LDSC analysis

We performed a post-GWAS genome-wide genetic correlation analysis for BF% and CVD-related traits using the LDSC software [30] by assuming that the effect size for each SNP in GWASs represents all SNPs in LD with it [17]. The genetic correlation of two traits (ranging from − 1 to 1) can be computed on LDSC software using the known LD structure of European ancestry reference data from the 1000 Genomes Project. Since low imputation quality may yield lower test statistics, we restricted our analyses to HapMap3 SNPs which seem to be well-imputed in most studies to minimize the bias in our analysis [17]. The mean chi-squared statistics in LDSC is higher in high-LD region compared with low-LD region and cannot be further analyzed when it is below 1.02. The analysis also provides a self-estimated intercept to show the sample overlap between single-trait GWASs [17].

### Partitioned genetic correlation analysis

The genetic correlation between BF% and CVDs was partitioned by 13 functional category using partitioned LDSC, including conserved region, DNaseI digital genomic footprinting region (DGF), DNase I hypersensitivity sites (DHSs), fetal DHSs, intron region, repressed region, super enhancers, transcription factor binding site (TFBS), transcribed region, and histone marks H3K4me1, H3K4me3, H3K9ac, and H3K27ac from Roadmap Epigenomics Project [31, 32]. We recalculated the LD scores of SNPs assigned to specific annotation which was used to evaluate the BF%-CVDs genetic correlation for each functional category. Additionally, we conducted another genetic correlation analysis partitioned by SNPs groups at different significant level [33]. The *P* value of a single SNP was defined a larger one in two GWASs, and then sorted and divided it into five groups in quartile, to further test the proportion of observed genetic correlation explained by each group and the robustness of our findings.

### Cross-trait meta-analysis

We applied MTAG [34], a novel approach for conducting meta-analysis of summary statistics from GWAS of multiple traits robust to sample overlap, to identify genome-wide significant loci between BF% and CVD-related traits [18], and detect novel genes by combining GWASs of two correlated diseases [35]. The key assumption of MTAG is that all SNPs share the same variance-covariance matrix of effect sizes among traits. As Turley et al. initially described in 2018, MTAG is a consistent estimator whose effect estimates always have a lower genome-wide mean-squared error than the corresponding single-trait GWAS, even if the assumption is not satisfied [18]. In addition, association statistics from MTAG also yield more statistical power and little inflation of the false discovery rate for each trait analyzed with high correlation, matching theoretical expectations [18].

### Fine-mapping credible set analysis

For each of the shared loci between BF% and HF or CAD that meet the cross-trait meta-analysis significance criteria, we extracted variants within 500 kb of the index SNP and then identified a 99% credible set of causal SNPs using the Bayesian likelihood fine-mapping algorithm [36, 37]. This algorithm only maps the primary signal and uses flat prior with steepest descent approximation to identify causal variants, which may reveal molecular mechanisms behind the associations. Details of the method have been described in previous studies [38, 39].

### Co-localization analysis

We first extracted summary statistics for variants within 500 kb of the index SNP at each of the shared loci between BF% and HF or CAD and then used R “coloc” package to perform genetic co-localization analysis to calculate the probability that the two traits shared a common genetic causal variant (H4) [40]. We conducted fully Bayesian co-localization analysis using the function “coloc.abf” in the R “coloc” package, which requires regression coefficients for each SNP and variance of these regression coefficients for each trait when only summary data are available [41]. In the present study, we considered loci with probability greater than 0.5 to be co-localized.

### GTEx TSEA

To test if shared gene sets were highly enriched or specific expressed in a tissue, we conducted a tissue-specific expression analysis (TSEA) [42, 43]. The analysis was based on the gene lists that were identified from cross-trait meta-analysis with a matching HUGO Gene Nomenclature Committee (HGNC) name. The gene expression data used in TSEA was collected using published RNA-Seq data from the GTEx project [44–46]. The raw GTEx data was derived from 189 post-mortem subjects consisting of 1839 samples from 45 different tissues. Considering the small sample size of GTEx data, we selected suggestive significant loci for this analysis (*P*_meta_ < 1 × 10^− 4^) to ensure the robustness of TSEA results. In addition, we used Benjamini–Hochberg correction to account for multiple testing [47].

### Over-representation enrichment analysis

To obtain biological insights for identified shared genes (*P*_meta_ < 5 × 10^− 08^) from cross-trait meta-analysis, we used the PANTHER tool to access enrichment of the gene sets in the Gene Ontology (GO) biological process and Reactome pathway [48, 49]. Benjamini–Hochberg procedure was used to account for multiple testing (false discovery rate < 0.05) [47].

### Bidirectional MR

We performed a bidirectional MR analysis between BF% and HF and CAD since they are genetically correlated, using inverse-variance weighted (IVW) as the primary method [50–52]. Median-based methods (simple and weighted), MR-Egger, MR-Robust Adjusted Profile Scores (MR-RAPS), and MR-Pleiotropy Residual Sum and Outlier (MR-PRESSO) methods were used as sensitivity analyses. The intercept of MR-Egger can be explained as a test of overall unbalanced horizontal pleiotropy [53, 54]. In addition, we also performed single-SNP and leave-one-out analysis to determine whether there was any single SNP that might drive the IVW point estimate. For instrumental variables, we have used the largest and latest GWASs for these traits [8–10]. We only selected independent genetic variants which are not in linkage disequilibrium (LD) (defined as *r*^2^ < 0.1) with other genetic variants based on European ancestry reference data from the 1000 Genomes Project. We chose the variant with the lowest *P* value for association with the exposure when genetic variants were in LD. For SNPs that were not available in outcome GWASs, we used the LD proxy search on the online platform (https://snipa.helmholtz-muenchen.de/snipa3/index.php/) to replace them with the proxy SNPs identified in high-LD (*r*^2^ > 0.8) or discard them if the proxies were not available.

### TWAS

To identify associations of BF% and CVDs with gene expressions in specific tissues, we conducted a TWAS using FUSION software package based on 44 post-mortal GTEx (version 6) tissue expression weights [19, 55, 56]. We applied Benjamini-Hochberg correction on TWAS *P* values of all gene-tissue pairs for each trait, and false discovery rate < 0.05 was considered significant [47].

## Results

### Genome-wide genetic correlation

We found strong positive genetic correlations with BF% for both HF (Rg = 0.47, *P* = 1.27 × 10^− 22^) and CAD (Rg = 0.22, *P* = 3.26 × 10^− 07^) in large study populations that were predominantly of European ancestry (more than 75%) (Table 1, Table S1). Additionally, we found nominally significant genetic correlation with BF% for ICH (Rg = 0.29; *P* = 0.021) and HDL (Rg = − 0.326; *P* = 0.048). However, we did not find evidence of genetic correlation between BF% and IS, MI, AF, TC, TG, and LDL (all *P* > 0.05) (Table 1). Estimates of SNP-based heritability on the observed scale using GWAS summary statistics are shown in Table S2.

**Table 1:** Genetic correlation between BF% and cardiovascular disease-related traits

Phenotype	Rg	Rg_SE	***P***
Coronary artery disease	0.223	0.044	3.26E−07
Heart failure	0.473	0.048	1.27E−22
Myocardial infarction	0.167	0.090	0.062
Intracerebral hemorrhage	0.292	0.126	0.021
Atrial fibrillation	0.068	0.053	0.199
Ischemic stroke	0.147	0.089	0.097
High-density lipoproteins	− 0.326	0.165	0.048
Low-density lipoproteins^a^	/	/	/
Total cholesterol^a^	/	/	/
Triglycerides	0.127	0.099	0.200

*Rg* genetic correlation estimate, *SE* standard error. ^a^Out of bound

**Fig. 2:**
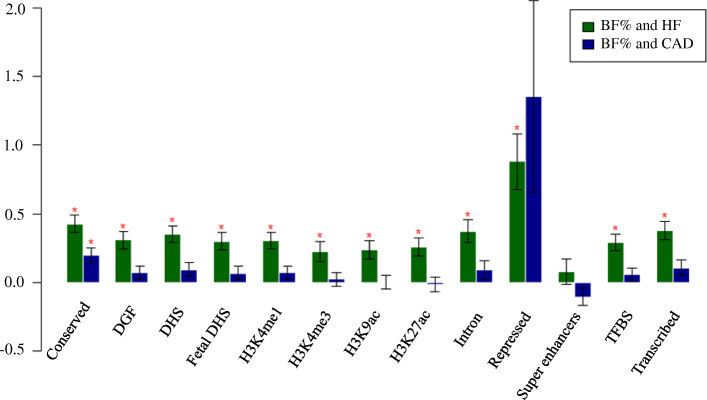
Partitioned genetic correlation between BF% and HF or CAD. The vertical axis represents the genetic correlation estimate Rg; the horizontal axis represents 13 functional categories. The asterisk represents significance (*P <* 0.05), error bars represent the standard error of genetic correlation estimates. DGF: DNaseI digital genomic footprinting; DHS: DNase I hypersensitivity site; TFBS: transcription factor binding sites

Since high positive genetic correlations between BF% and CVDs was only observed with HF and CAD, we further used 13 functional annotations to evaluate genetic correlations between BF% and HF or CAD in the partitioned genetic correlation analysis by specific functional category. The highest magnitude of significant genetic correlation was in the repressed region (Rg = 0.88; *P* = 1.51 × 10^− 05^) for BF% and HF, which could restore the phenotypic effects of a mutant gene. The correlation estimate for BF% and CAD was highest in the conserved region (Rg = 0.20; *P* = 5.00 × 10^− 04^), where this region remained almost unchanged during evolution (Fig. 2; Table S3). The shared genetic etiology for BF% and CVDs encourages the exploration of a common pathophysiology, especially in specific functional categories. Subsequently, we conducted a partitioned genetic correlation analysis by SNP groups with different *P* values and found that the first two groups remained significant (*P* < 1.00 × 10^− 05^), suggesting that our findings were robust (Figure S1-S2).

### MTAG for single traits

**Fig. 3:**
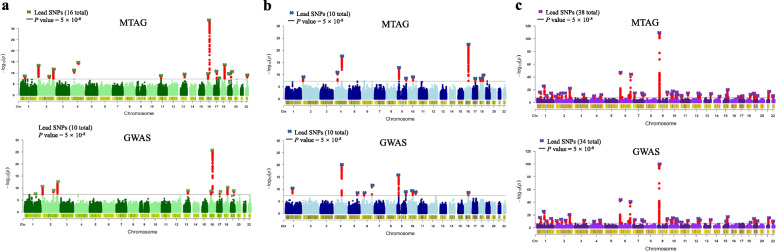
The up and down plots display the MTAG and GWAS results for **a** BF%, **b** HF, and **c** CAD, respectively, for a fixed set of SNPs. The *x* axis shows chromosomal position, and the *y* axis shows significance on a −log10 scale. The line marks the threshold for genome-wide significance (*P* = 5 × 10^− 8^). Each approximately independent genome-wide significant association (lead SNP) is marked by a cross

Manhattan plots from the GWAS and MTAG analyses for each trait are shown in Fig. 3. From GWAS to MTAG, the total number of lead SNPs increased from 11 to 16 for BF% and from 42 to 51 for CAD, while no changes were found for HF. We confirmed most of the previously identified loci and found novel associations between BF% and HF and CAD. Out of the 16 independent loci reported for BF% using MTAG, 6 were novel associations and 2 of these fell within protein-coding gene bodies (*PLA2G6*, *RPTOR*) (Table S4). Among the 51 loci associated with CAD, 13 were novel loci, 10 of which were mapped to protein-coding genes (i.e., *HNRNPUL1*, *NAA25*, *FES*, *TNS1*, *CYP46A1*, *ABCG8*, *IGF2BP1*, *BCAS3*, *SMG6*, *APOE*) (Table S4). The proteins encoded by these gene and gene-related pathways are shown in Table S4. Out of the 10 independent loci identified for HF, 8 were novel, 3 of which were mapped to protein-coding genes (i.e., *FTO*, *NPC1*, *IGF2BP1*) (Table S4).

### Cross-trait meta-analysis

**Fig. 4:**
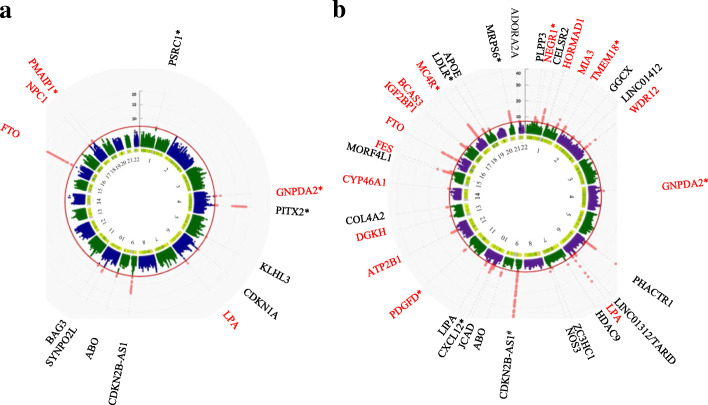
Circus Manhattan plot of cross-trait meta-analysis. The first layer of the plot illustrates the chromosome position and the second layer illustrates the representative genes of significant loci. Genes in red are shared genes between two traits [**a** BF% and HF; **b** BF% and CAD] with single-trait *P* value < 0.05. The inside layer illustrates the significance level − log10 (*P* value) shared markers from cross-trait meta-analysis. The red dots indicate genome-wide significant (*P* < 5 × 10^− 8^). Genes at loci in close proximity were assigned one gene label separated by a slash. Asterisks represent the gene closest to index SNP. #: The *P* value of gene CDKN2B-AS1 (rs4977574) is 9.9 × 10^−84^ (out of range)

Given the strong genetic correlation between BF% and HF or CAD, we further used MTAG to perform genome-wide meta-analysis to improve our power to identify shared significant genetic loci (meta-analysis *P* < 5 × 10^− 08^ and single trait *P* < 0.05). The genomic control parameter (*λ*) was 1.16 in the cross-trait meta-analysis for BF% and HF, while *λ* = 1.20 for BF% and CAD (Figure S3-S4). Illustrative calculations in the two-trait setting were 0.0012 for BF% and CAD and 0.0035 for BF% and HF. The Manhattan plot of these results indicated that shared genetic loci drove the overall significance of the meta-analysis (Fig. 4).

We identified 5 genome-wide significant independent loci for BF% and HF (Table 2). Out of the 5 independent loci, 3 were mapped to protein-coding genes (i.e., *FTO*, *NPC1*, *LPA*). The strongest signal was observed on chromosome 16 at the *FTO* region (index SNP rs9937053, *P*_meta_ = 1.09 × 10^− 18^), the first GWAS-identified susceptibility gene for obesity [57, 58]. This locus was not only significant after meta-analysis, but also reached genome-wide significance in both single-trait GWAS of BF% (*P* = 9.86 × 10^− 26^) and HF (*P* = 2.99 × 10^− 08^). The second strongest signal was in close proximity to an intergenic region closest to the *GNPDA2* gene on chromosome 4 (index SNP rs10938397, *P*_meta_ = 4.36 × 10^− 10^), a critical gene involved in lipid and glucose metabolism. Previous studies found that the expression level of *GNPDA2* alters the transcriptome profile of human adipose-derived mesenchymal stem cells [59]. In addition, we found that genetic loci represented by rs10455872 (*P*_meta_ = 3.88 × 10^− 09^) on *LPA* are associated with both BF% and HF after meta-analysis. Variation within *LPA* has shown strong association with Lp(a)-cholesterol levels, which is an independent risk factor for cardiovascular-related events [60].

**Table 2:** Genome-wide significant loci by cross-trait meta-analysis at sentinel SNPs associated with BF% and HF (*P*_meta_ < 5 × 10^− 8^; single trait *P* < 0.05)

SNP	CHR	Position	Ref. allele	Alt. allele	***P***_**BF%**_	***P***_**HF**_	***P***_**META**_	Variant annotation	Genes within clumping region
rs9937053	16	53799507	G	A	9.858E−26	2.992E−08	1.09E−18	Intron	AKTIP,CHD9,FTO,FTO-IT1,LOC643802,LOC102723373,RBL2,RPGRIP1L
rs1652348	18	21147509	C	T	0.0006127	2.477E−06	3.35E−08	Intron	ANKRD29,C18orf8,CABLES1,LAMA3,LOC102724246,NPC1,RIOK3,TMEM241.TTC39C
rs7234864	18	57734857	C	T	6.827E−07	1.804E−05	8.159E−09	Regulatory region	CCBE1,MC4R,PMAIP1
rs10938397	4	45182527	A	G	1.359E−07	2.948E−06	4.36E−10	Intergenic	GNPDA2,GUF1
rs10455872	6	161010118	A	G	0.02079	1.892E−11	3.879E−09	Intron	IGF2R,LOC729603,LPA,LPAL2,MAP 3 K4,PLG,SLC22A1,SLC22A2,SLC22A3

*SNP* single-nucleotide polymorphism, *CHR* chromosome

The cross-trait meta-analysis between BF% and CAD identified 16 genome-wide significant loci (Table 3), 10 of which were in protein-coding gene bodies (i.e., *LPA*, *HORMAD1*, *MIA3*, *WDR12*, *DGKH*, *CYP46A1*, *FES*, *FTO*, *IGF2BP1*, *BCAS3*). The most significant locus is characterized by the *LPA* gene (index SNP rs10455872, *P*_meta_ = 3.14 × 10^− 28^). The second locus (index SNP rs8050136, *P*_meta_ = 4.02 × 10^− 19^) was mapped to the *FTO* gene. These loci were also found to be significant in the meta-analysis for BF% and HF, showing genetic overlaps between BF% and CVD-related traits. The third strongest signal was observed closest to the *MC4R* gene (index SNP rs663129, P_meta_ = 3.10 × 10^− 18^), which is involved in the leptin signaling pathway and its disruption is a causal factor of obesity [61].

**Table 3:** Genome-wide significant loci by cross-trait meta-analysis at sentinel SNPs associated with BF% and CAD (*P*_meta_ < 5 × 10^− 8^; single trait *P* < 0.05)

SNP	CHR	Position	Ref. allele	Alt. allele	***P***_**BF%**_	***P***_**CAD**_	***P***_**META**_	Variant annotation	Genes within clumping region
rs2590942	1	72885281	T	G	4.98E−07	1.73E−03	6.166E−09	Intergenic	NEGR1
rs4970926	1	150673684	T	C	1.43E−03	3.99E−05	4.269E−08	Intron	ADAMTSL4,ANP32E,ANXA9,APH1A,ARNT,BNIPL,C1orf51,C1orf54,C1orf56,CA14,CDC42SE1,CTSK,CTSS,ECM1,ENSA,FAM63A,GOLPH3L,HORMAD1,KIAA0460,LASS2,LYSMD1,MCL1,MLLT11,MRPS21,PIP5K1A,PRPF3,PRUNE,RP11-68I18.1,SCNM1,SEMA6C,SETDB1,TARS2,TMOD4,TNFAIP8L2,VPS72
rs2133189	1	222814442	C	T	1.99E−02	2.42E−12	7.714E−09	Intron	AIDA,C1orf58,DISP1,FAM177B,HHIPL2,MIA3,TAF1A,TLR5
rs13396935	2	653195	G	A	1.42E−09	6.43E−04	2.493E−11	Regulatory Region	ACP1,FAM150B,LOC391343,SH3YL1,SNTG2,TMEM18
rs6435169	2	203753016	G	A	4.61E−02	7.54E−18	7.068E−10	Intron	ABI2,ALS2CR8,ALS2CR13,BMPR2,CYP20A1,ICA1L,NBEAL1,WDR12
rs10938397	4	45182527	A	G	1.36E−07	1.06E−03	1.176E−09	Intergenic	GNPDA2,GUF1
rs10455872	6	161010118	A	G	2.08E−02	5.73E−39	3.141E−28	Intron	IGF2R,LPA,LPAL2,MAP 3K4,PLG,SLC22A1,SLC22A2,SLC22A3
rs11226029	11	103693627	G	A	3.54E−02	1.14E−09	2.967E−11	Intron	DDI1,DYNC2H1,PDGFD
rs4842662	12	89933446	T	C	3.00E−05	7.13E−07	1.861E−11	Intron	ATP2B1,DUSP6,GALNT4,WDR51B
rs9532984	13	42634693	G	A,C,T	1.93E−02	1.62E−06	1.802E−08	Intron	AKAP11,DGKH,KIAA0564
rs3752958	14	100182687	C	A T	2.52E−02	7.98E−07	1.42E−08	Intron	BCL11B,CCNK,CYP46A1,DEGS2,EML1,EVL,HHIPL1,SETD3
rs1894400	15	91428955	C	T	1.95E−02	1.54E−07	1.118E−09	Intron	BLM,CRTC3,FES,FURIN,HDDC3,IQGAP1,MAN2A2,PRC1,RCCD1,SV2B,UNC45A,VPS33B
rs8050136	16	53816275	C	A	1.36E−25	4.65E−03	4.023E−19	Intron	AKTIP,CHD9,FTO,RBL2,RPGRIP1L
rs9906944	17	47091420	C	G,T	2.86E−08	1.17E−05	1.935E−12	Intron	ABI3,ATP5G1,B4GALNT2,C17orf92,CALCOCO2,GIP,GNGT2,HOXB1,HOXB2,HOXB3,HOXB4,HOXB5,HOXB6,HOXB7,HOXB8,HOXB9,HOXB13,IGF2BP1,NGFR,PHB,PHOSPHO1,SNF8,TTLL6,UBE2Z,ZNF652
rs4456565	17	58921974	T	C	4.22E−02	6.63E−07	3.104E−08	Intron	APPBP2,BCAS3,C17orf64,LOC729617,PPM1D,USP32
rs663129	18	57838401	G	A	1.47E−10	3.20E−08	3.097E−18	Intergenic	CCBE1,MC4R,PMAIP1

*SNP* single-nucleotide polymorphism, *CHR* chromosome.

### GTEx TSEA

**Fig. 5:**
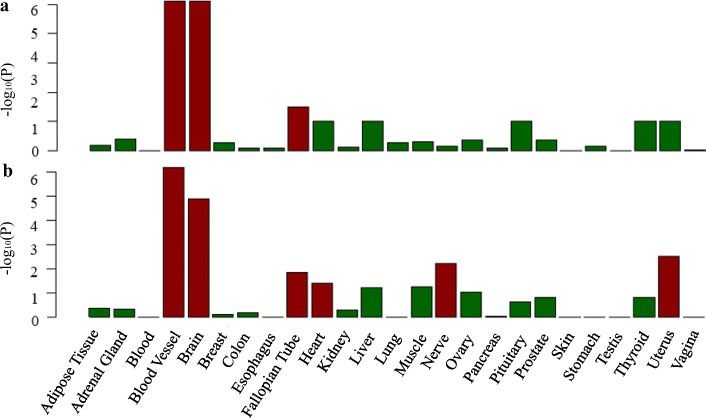
GTEx tissue enrichment analysis for expression of cross-trait-associated genes (*P*_meta_ < 1 × 10^− 4^) for BF% and HF (**a**) and BF% and CAD (**b**). Red represents significant tissue enrichment after Benjamin-Hochberg correction

In order to assess whether shared genes between BF% and CVDs are enriched for expression in the disease-relevant tissue, we conducted the TSEA using the GTEx pilot data. We found that shared genes of BF% and HF had three significantly enriched tissues, including blood vessel, brain, and fallopian tube (Fig. 5). The shared genes of BF% and CAD were enriched in six tissues including blood vessel, brain, fallopian tube, heart, nerve, and uterus (Fig. 5). The most strongly enriched tissue for both BF% and HF or CAD was part of the cardiovascular and nervous system (Fig. 5).

### Over-representation enrichment analysis

The GO analysis indicated several significant shared biological processes between BF% and HF or CAD (false discovery rate < 0.05) such as glutathione derivative metabolic, glutathione derivative biosynthetic, and nitrobenzene metabolic processes (Table S5-S6). In additional analyses of Reactome pathways, we found that shared association signals for BF% and HF or CAD were significantly enriched in glutathione conjugation and plasma lipoprotein-related pathways (Table S7-S8). In general, the identified shared genes between BF and CVDs showed common significant enrichment in expression for glutathione metabolic-related pathways.

### Fine-mapping and co-localization analysis

Lists of credible set SNPs in each shared locus for BF% and HF or CAD from fine mapping are shown in Table S9-S10. The co-localization analysis showed that 4 out of 5 loci and 16 out of 16 share causal variants between BF% and HF or CAD, respectively (Table S11-S12). The results of co-localization analysis were consistent with the cross-trait meta-analysis.

### Single-trait TWAS

**Fig. 6:**
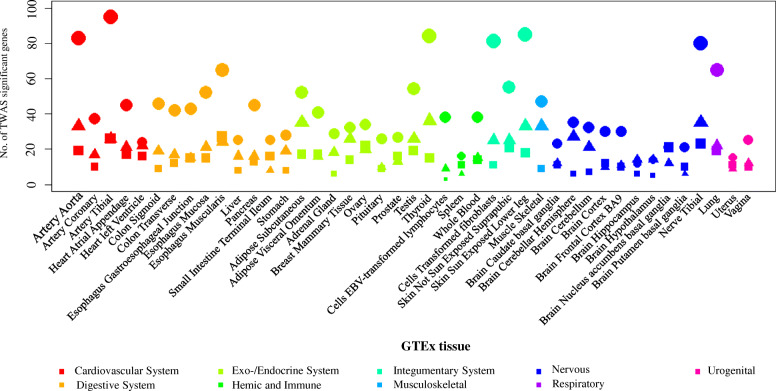
Number of significant expression-trait associations from transcriptome-wide association study (TWAS). The total number is the significant expression-trait associations after Bonferroni correction (false discovery rate < 0.05). The square represents BF%, the circle represents CAD, and the triangle represents HF. GTEx: genotype-tissue expression project

We conducted a TWAS analysis to examine if there are genes whose expression are related with BF%, CAD, and HF, and to determine if these genes are common among these traits. A total of 270 gene-tissue pairs were found across 44 GTEx tissues to be significantly associated with BF% after Benjamini-Hochberg correction (false discovery rate < 0.05) [47], in addition to 786 gene-tissue pairs with CAD, and 270 gene-tissue pairs with HF (Fig. 6). Among these gene-tissue pairs, 32 overlapped between BF% and HF, most of which were observed in nervous and cardiovascular system (Table S17). Notably, *C18orf8* and *NPC1* are expressed in multiple tissues including brain, nerve, artery, and adipose tissue. Consistent with a previous study [62], *NPC1* was validated as a shared genetic component in lipid metabolism and cardiovascular health from our TWAS results. In addition, the methylation levels of *C18orf8* were considered as an epigenetic mechanism in maternal obesity and early life origins of CVD and cancers [63]. Furthermore, we identified 28 gene-tissue pairs that were shared by BF% and CAD (Table S17). Most of the associations were found in exo-/endocrine and digestive system. Some genes are expressed among multiple tissues such as *ATP5G1*, *SLC22A3*, *SNF8*, and *UBE2Z*. *ATP5G1* has been shown to encode a subunit of mitochondrial ATP synthase [64] and *SLC22A3* have been reported to be expressed at the blood-brain barrier and alters neuronal excitability [65]. By integrating human and mouse results, previous studies have predicted that *SNF8* and *UBE2Z* play a causal role in the development of CAD through a role in the vasculature [66, 67].

### Bidirectional MR

We conducted a bidirectional MR analysis to evaluate potential causal associations between BF% and HF and CAD. To assess the causal effect of BF% on HF and CAD, we used 10 previously identified genetic variants for BF% (Table S13). To assess the causal effect of HF and CAD on BF%, 5 and 24 previously identified genetic variants were used as instruments for HF and CAD, respectively (Table S14). We found that genetically predicted BF% was causally associated with an increased risk of HF (OR 1.63 per 1-SD increase in BF%, *P* = 4.16 × 10^− 04^) (Table 4) after Bonferroni correction. However, HF was not causally associated with BF%. In addition, there were no causal relationships between BF% and the risk of CAD. Sensitivity analysis showed that our results were reliable and were not affected by horizontal pleiotropy or outliers (Table 4, Table S15-S16).

**Table 4:** The bidirectional MR analysis of BF% levels and CVD traits

Outcome	MR methods	Forward			Reserved		
		Causal effect size	SE	***P*** value	Causal effect size	SE	***P*** value
HF	IVW	0.488	0.138	4.16E−04	− 0.036	0.094	0.71
	SMB	0.451	0.134	7.45E−04	− 0.067	0.142	0.64
	WMB	0.456	0.132	5.46E−04	0.027	0.118	0.82
	MR-RAPS	0.594	0.088	1.22E−11	− 0.036	0.099	0.72
	MR-PRESSO	0.488	0.138	6.42E−03	−0.036	0.077	0.67
	MR-Egger	1.452	0.571	0.03	0.323	0.290	0.35
CAD	IVW	0.264	0.330	0.42	0.014	0.051	0.78
	SMB	0.437	0.175	0.01	− 0.010	0.043	0.81
	WMB	0.491	0.165	2.93E−03	0.025	0.042	0.55
	MR-RAPS	0.563	0.096	3.88E−09	0.008	0.028	0.78
	MR-PRESSO	0.264	0.330	0.44	0.014	0.051	0.78
	MR-Egger	− 0.152	1.617	0.93	− 0.013	0.115	0.91

*SE* standard error, *IVW* inverse-variance weighted, *SMB* simple median, *WMB* weighted median, *RAPS* robust adjusted profile scores, *PRESSO* pleiotropy residual sum and outlier

## Discussion

Using large-scale GWAS data from international consortia, we analyzed shared genetic etiology and causal associations between BF% and CVD-related traits. First, we found strong positive genetic correlations and further identified novel shared genetic loci between BF% and HF or CAD. Second, we found that the shared genetic loci were enriched mainly in blood vessels and brain tissues and glutathione-related metabolic pathway using functional analysis. Third, we found that the shared genes between BF% and HF or CAD are mostly from nervous, cardiovascular and exo-/endocrine system using TWAS analysis. Finally, we found that BF% was causally associated with HF using bidirectional MR analysis results. Taken together, evaluation of the genetic correlation and causality between BF% and CVDs furthers the understanding of the shared loci and biological mechanisms underlying this comorbidity.

BF% is becoming recognized as a more predictive measurement in cardiovascular risk assessment than BMI or waist circumference, and increased BF% was often related to higher CVD risks with the presence of the metabolic abnormalities [25, 68]. Genetic studies have suggested that most of the BF% loci also affect cardiometabolic traits and comorbidities [8]. Compared to GWAS results for single traits, MTAG discovered 6 novel loci for BF%, 8 novel loci for HF, and 13 novel loci for CAD. Importantly, we further conducted genome-wide cross-trait meta-analysis to improve our power to identify specific shared loci, including 3 novel loci between BF% and HF and 8 novel loci between BF% and CAD which did not reach genome-wide significance in previous GWAS. We highlight the potentially interesting functions of the novel associations for *NPC1*, *PMAIP1*, and *GNPDA2* between BF% and HF, along with *NEGR1*, *HORMAD1*, *GNPDA2*, *DGKH*, *CYP46A1*, *FES*, *POC1B*, and *BCAS3* between BF% and CAD. Most of these loci have significant single-tissue expression quantitative trait loci (eQTL), mainly in the cardiovascular, nervous, and exo-/endocrine system (Table S18-S19).

The only top loci common to the BF%-HF and BF%-CAD meta-analysis was rs10938397 near *GNPDA2*, which encodes an enzyme that catalyzes the deamination of the glucosamine-6-phosphate involved in the hexosamine signaling pathway [59]. An animal study also suggested that *GNPDA2* was involved in the regulation of body weight, fat and energy metabolism in the development of cardiovascular disease [69]. Other top associations for BF%-HF were *NPC1* gene variations, which might lead to metabolic diseases by modulating steroid hormone synthesis and lipid homeostasis [70]. *NPC1* is known to play a critical role in the atherosclerotic progression, and loss-of-function mutations in *NPC1* can cause adiposity in humans [71, 72]. In particular, the *NPC1* gene was also confirmed to be shared between BF% and HF using TWAS (Table S17). BCAS3 is a cytoskeletal protein that promotes directional cell migration and angiogenesis in vitro and is implicated in CAD [73]. Additionally, other causal genes are highly expressed or known to act in human brain function (i.e., *NEGR1*, *GNPDA2*, *CYP46A1*, *DGKH*) [74–76] and various carcinomas (i.e., *PMAIP1*, *HORMAD1*, *FES*, *BCAS3*) [77–79], indicating the potential role of metabolic dysregulation in the pathogenesis of obesity and cardiac events.

We found that the shared loci for BF% with HF and CAD were highly expressed in blood vessel/heart tissues, indicating that these traits might be caused by dysfunction of the cardiovascular system. We found that the *FTO* gene on chromosome 16 was associated with both BF%/HF and BF%/CAD. *FTO* is an essential regulator in the development of obesity-induced metabolic and vascular changes, which is independent of its known function in regulation of obesity [80]. Previous studies found that *FTO* plays a critical role in cardiac contractile function during homeostasis, remodeling, and regeneration for HF and CAD [81, 82]. Moreover, from the perspective of gene function, overlaps in genetic and molecular pathways advance our understanding of the potential role of brain/nerve tissue diseases in the association between BF% and CVDs. For example, the *NEGR1* gene, which is strongly expressed in the brain, has been reported to affect neuronal control of food intake and promote obesity [83]. People who have mutations in the *NEGR1* gene may have abnormal fat deposition in various peripheral cells, suggesting a potential molecular target for regulating body fat [84]. In the over-representation enrichment analysis, glutathione-related metabolic pathway was found to be common in shared genes of both BF%/HF and BF%/CAD meta-analyses. Experimental studies have reported that glutathione, which is the most abundant antioxidant in the heart, plays a key role in preventing the damage of redox homeostasis to cause obesity-related cardiovascular complications [85, 86]. More importantly, the protective effect of glutathione on blood vessel and brain tissue against oxidative stress has been previously reported and partially explains our findings [87, 88]. However, the nonsignificant genetic correlations between BF% and the other CVD-related traits suggested heterogeneous genetic architecture among CVD-related traits, which cannot be determined without further study.

In addition to the cardiovascular and nervous system, our TWAS also reported tissues enrichment from the exo-/endocrine and digestive system suggesting that the shared pathway between BF% and CVDs might have significant functions extending beyond brain and blood vessels. Besides a variety of adaptations/alterations in cardiac structure and function, body fat may affect the risk of HF with an altered metabolic profile in exo-/endocrine system [89]. The incidence of CVDs are known to be closely related to endocrine factors such as hormone levels in vivo, which may be altered by body fat distribution and then affect the whole body organs such as ovary, thyroid, colon, and liver. For example, adolescent girls with obesity and polycystic ovary syndrome have increased fasting and postprandial plasma triglycerides and ApoB-lipoprotein remnants, and these indices are highly associated with early subclinical CVD risk [90]. Indeed, an essential cause of CVDs is excessive body fat, which can be distributed in various organs of the body and cause pathological changes there. Thus, TWAS provided additional evidence of the enrichment of BF% and CVD genes expressed are not specific to a certain tissue; rather, it seems to be generalizable across metabolic organs of the whole body.

The bidirectional MR analysis showed a significant association between genetically predicted BF% and increased risk of HF (1.63-fold risk per 1-SD increase in BF%), which was stronger than that observed in a community-based cohort study (*n* = 100,280) at 1.32-fold per 1-SD increase in BF% [91]. Our findings were also consistent with a large cohort study which was conducted in 5520 community-based, elderly individuals, showing that higher BF% might contribute to the development of HF through increasing ventricular-arterial stiffness [92]. However, clinically, the association between BF% and HF could be bidirectional. In addition, previous studies suggested that BMI or body weight was positively or negatively correlated with HF or clinical outcomes [93, 94]. Although various associations were observed between BF% and CVDs, they were inconsistent possibly due to reverse causation and confounding. Therefore, this MR design is warranted to obtain causality which largely avoided bias such as reverse causation and confounding under MR key assumptions. Our MR findings provided reliable evidence of a causal role of BF% in HF based on large GWAS consortia regardless of unknown confounders existing in observational studies, highlighting the benefit of targeting BF% in the prevention of HF. The potential mechanisms underlying the causal association between BF% and HF require investigation, but the shared loci and related pathway could provide new directions in revealing shared etiologies.

In addition, we observed nominal positive correlation of BF% with intracerebral hemorrhage and negative correlation with HDL from genetic correlation analysis, but not with any other lipid profiles. Previous studies have suggested that low HDL may contribute directly to establishing or maintaining the obese condition due to the role of HDL in cellular lipid transport [95]. HDL contribute to modulating body fat content by controlling the extent of lipolysis in mice model, which appears to be key components of lipid metabolism in adipose tissue and constitute new therapeutic targets in obesity [96]. In cell culture studies, HDL specifically increased catecholamine-induced lipolysis possibly through modulating the adipocyte plasma membrane cholesterol content [96]. In addition, the HERITAGE Family Study has shown that high HDL cholesterol levels are good correlates of the metabolic profile, representing a better index in CVD risk assessment [97].

This study had limitations. First, the study is limited by the quality of data collected including the BF% measurement, CAD diagnosis, medication administration, and so on. However, each previous study has conducted the study-specific quality control to ensure the data quality. In addition, considering the natural and random assortment of genetic variants during meiosis yielding the random distribution of genetic variants in populations, the result and conclusion of this genetic analysis are less likely to be influenced by confounding factors. Second, while BF% accurately reflects the proportion of fat content in the human body, it does not give full insight to distinguish between visceral and subcutaneous fat or fat content in each anatomical part. Further studies are required to explore the genetic association of body fat distribution with CVD risk. Third, we used limited number of SNPs as instrumental variables in the MR analysis, so we cannot exclude the possibility that our findings might have been affected by weak instrument bias, although all genetic instruments were associated with the exposure (F-statistic> 10). Fourth, this study did not assess sex-specific genetic effects using LDSC and MR analysis. Since we conducted the analysis using summary-level GWASs without individual data, it is difficult for us to verify the findings of this study for any variation with sex-specific analysis. Fifth, we could not re-test our hypothesis using additional or alternative large cohort without individual information in the present study. However, the data we used are the largest and latest GWASs for these traits, and the sample sizes of other cohorts are small from which we cannot yield more reliable results. Finally, the current study was limited to assessing shared genetic factors between BF% and CVD-related traits, which could only explain a small proportion of these traits. The effect of gene-environment interaction, in which the genetic variants lead to the occurrence of diseases in high-risk environments, may explain part of the rest variance [98]. Although CAD and MI or stroke vest in similar pathophysiologic mechanisms, the complex roles of interactions between genes and environmental factors may be different [99]. More large-scaled, well-conducted studies on gene-environment interaction in the development of CVDs are warranted.

## Conclusions

In summary, our findings provide strong evidence of genetic correlations between BF% and CVDs, including HF and CAD. Additionally, we found a causal association between BF% and HF, which further supports targeted reduction of adiposity for improved cardiovascular outcomes. These results advance our understanding of body fat and provide novel insight into the common genetic basis of BF% and CVDs from molecular and functional levels. Notably, we identified novel genetic loci in both single-trait GWAS and cross-trait meta-analysis for multi-trait GWASs using MTAG. This work reinforces the idea that BF% and CVDs share common biological processes and opens up a new way for early prevention of CVDs.

## Supplementary Information

**Additional file 1: Table S1.** Summary of GWAS data. **Table S2.** SNP based heritability estimated by LDSC. **Table S3.** Partitioned genetic correlation between BF% and 2 cardiac traits. **Table S4.** Novel loci in MTAG for single trait compared with GWAS. **Table S5.** GO biological process pathway analysis for BF% and HF. **Table S6.** GO biological process pathway analysis for BF% and CAD. **Table S7.** Reactome pathways analysis for BF% and HF. **Table S8.** Reactome pathways analysis for BF% and CAD. **Table S9.** List of credible set SNPs in each locus from fine mapping. For each of the 5 BF% and HF shared loci, the table lists all SNPs within 500 kb in the 99% credible sets that were calculated. **Table S10.** List of credible set SNPs in each locus from fine mapping. For each of the 16 BF% and CAD shared loci, the table lists all SNPs within 500 kb in the 99% credible sets that were calculated. **Table S11.** Genome-wide significant loci by cross-trait meta-analysis at sentinel SNPs associated with BF% and HF with directional effect (Pmeta < 5×10-8; single trait *P* < 0.05). **Table S12.** Genome-wide significant loci by cross-trait meta-analysis at sentinel SNPs associated with BF% and CAD with directional effect (Pmeta < 5×10-8; single trait P < 0.05). **Table S13.** Specific characteristics of genetic instruments of MR analysis of BF% levels upon CVD traits. **Table S14.** Specific characteristics of genetic instruments of MR analysis of CVDs upon BF% levels. **Table S15.** MR analysis between BF% and cardiac traits in a leave one out approach. **Table S16.** MR analysis between BF% and cardiac traits for a single snp. **Table S17.** Significant overlap transcriptome-wide association analysis results between BF% and CVDs. (FDR<0.05). **Table S18.** Significant single-tissue eQTL from GTEx for 3 representative gene between BF% and HF. **Table S19.** Significant single-tissue eQTL from GTEx for 8 representative gene between BF% and CAD.

**Additional file 2: Figure S1.** Partitioned genetic correlation between BF% and HF by genetic variants groups with different *P* value. **Figure S2.** Partitioned genetic correlation between BF% and CAD by genetic variants groups with different P value. **Figure S3.** QQ plot of cross-trait meta-analysis between BF% and HF. **Figure S4.** QQ plot of cross-trait meta-analysis between BF% and CAD.

## Data Availability

All data used in the present study were obtained from genome-wide association study summary statistics which were publicly released by genetic consortia.

## Abbreviations

AF: Atrial fibrillation
BF%: Body fat percentage
BMI: Body mass index
CAD: Coronary artery disease
CARDIoGRAM: Coronary ARtery Disease Genome wide Replication and Meta-analysis
CNS: Central nervous system
CVD: Cardiovascular disease
DGF: DNaseI digital genomic footprinting region
DHS: DNase I hypersensitivity sites
GERFHS: Genetic and Environmental Risk Factors for Hemorrhagic Stroke
GIANT: Genetic Investigation of ANthropometric Traits
GLGC: Global Lipids Genetics Consortium
GOCHA: Genetics of Cerebral Hemorrhage with Anticoagulation
GTEx: Genotype-Tissue Expression
GWAS: Genome-wide association study
HDL: High-density lipoprotein
HERMES: Heart Failure Molecular Epidemiology for Therapeutic Targets
HF: Heart failure
HGNC: HUGO Gene Nomenclature Committee
ICH: Intracerebral hemorrhage
IS: Ischemic stroke
ISGC: International Stroke Genetics Consortium
IVW: Inverse-variance weighted
LD: Linkage disequilibrium
LDL: Low-density lipoprotein
LDSC: Linkage disequilibrium score regression
MI: Myocardial infarction
MR: Mendelian randomization
MTAG: Multi-trait analysis of GWAS
PRESSO: Pleiotropy Residual Sum and Outlier
RAPS: Robust Adjusted Profile Scores
SiGN: NINDS Stroke Genetics Network
SNP: Single-nucleotide polymorphism
TC: Total cholesterol
TFBS: Transcription factor binding site
TG: Triglycerides
TSEA: Tissue-specific expression analysis
TWAS: Transcriptome-wide association study
