# Supplementary material for: Shared genetic etiology and causality between body fat percentage and cardiovascular diseases: a large-scale genome-wide cross-trait analysis
Source: BMC Med. 2021 Apr 29;19:100. doi: 10.1186/s12916-021-01972-z (PMC8082910; doi:10.1186/s12916-021-01972-z)
Supplement: Supplementary file 2 — Additional file 2: Figure S1. Partitioned genetic correlation between BF% and HF by genetic variants groups with different P value. Figure S2. Partitioned genetic correlation between BF% and CAD by genetic variants groups with different P value. Figure S3. QQ plot of cross-trait meta-analysis between BF% and HF. Figure S4. QQ plot of cross-trait meta-analysis between BF% and CAD. [file 12916_2021_1972_MOESM2_ESM.pptx]

## Slide 1
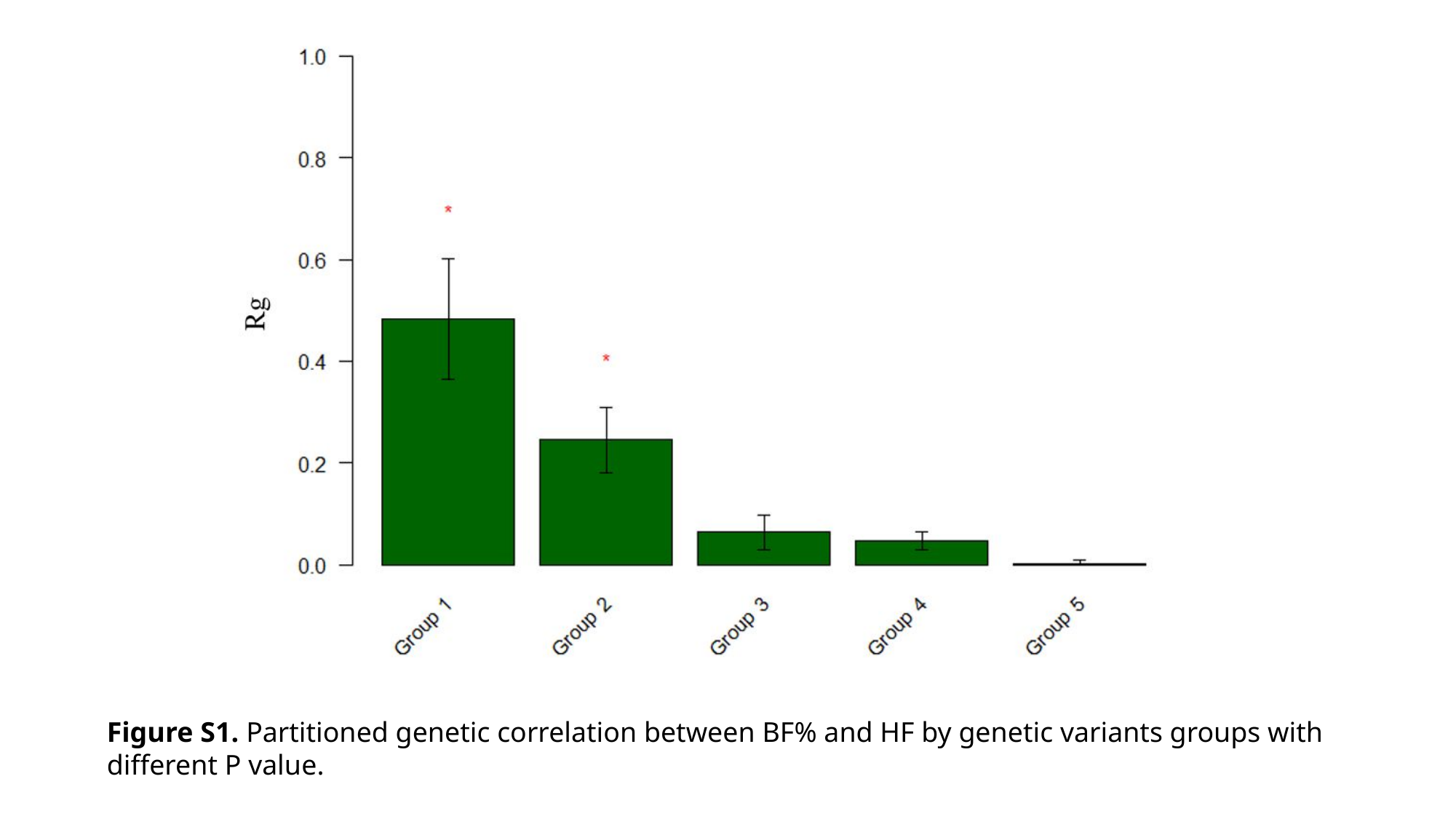

Figure S1. Partitioned genetic correlation between BF% and HF by genetic variants groups with different P value.

## Slide 2
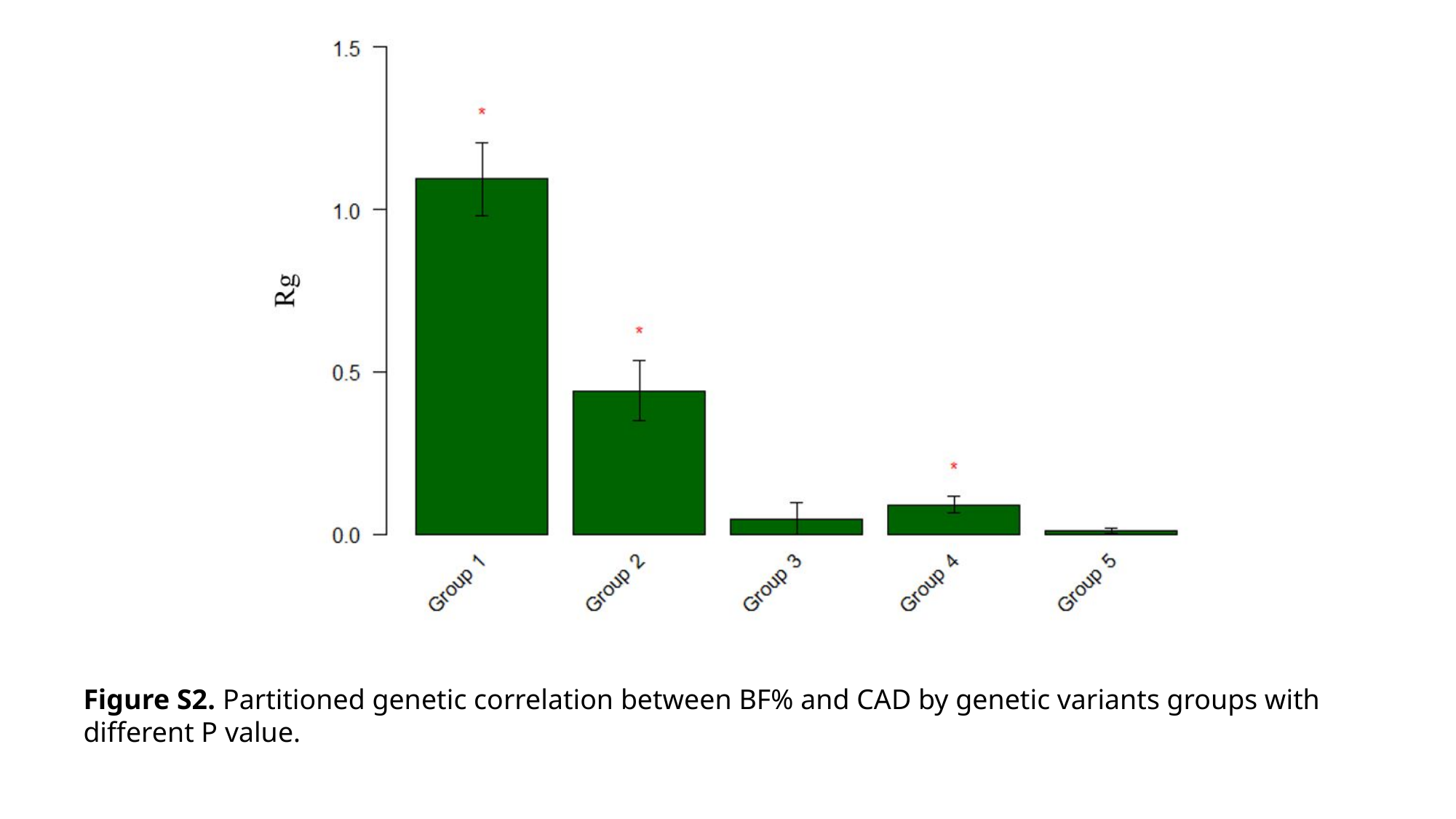

Figure S2. Partitioned genetic correlation between BF% and CAD by genetic variants groups with different P value.

## Slide 3
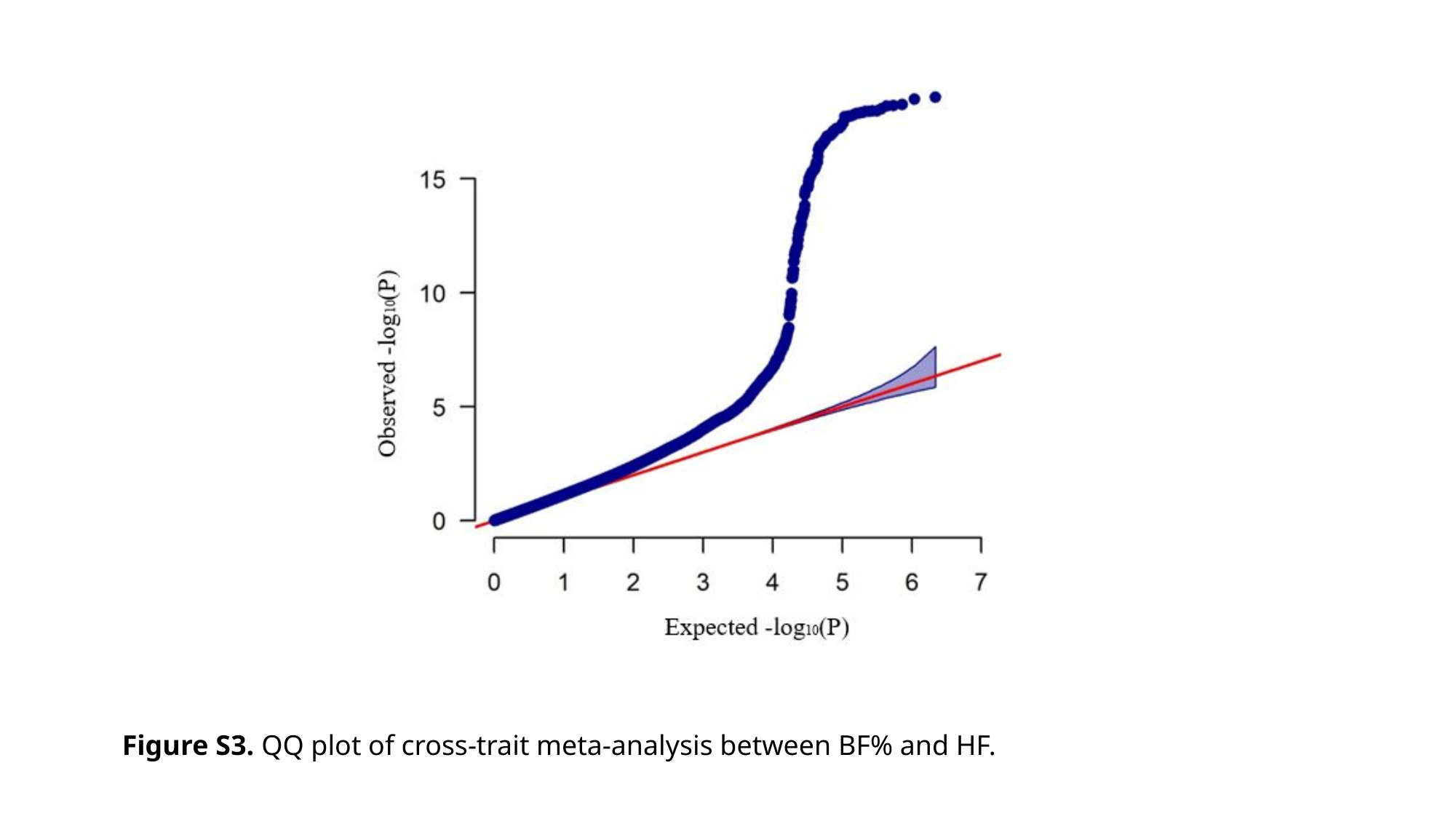

Figure S3. QQ plot of cross-trait meta-analysis between BF% and HF.

## Slide 4
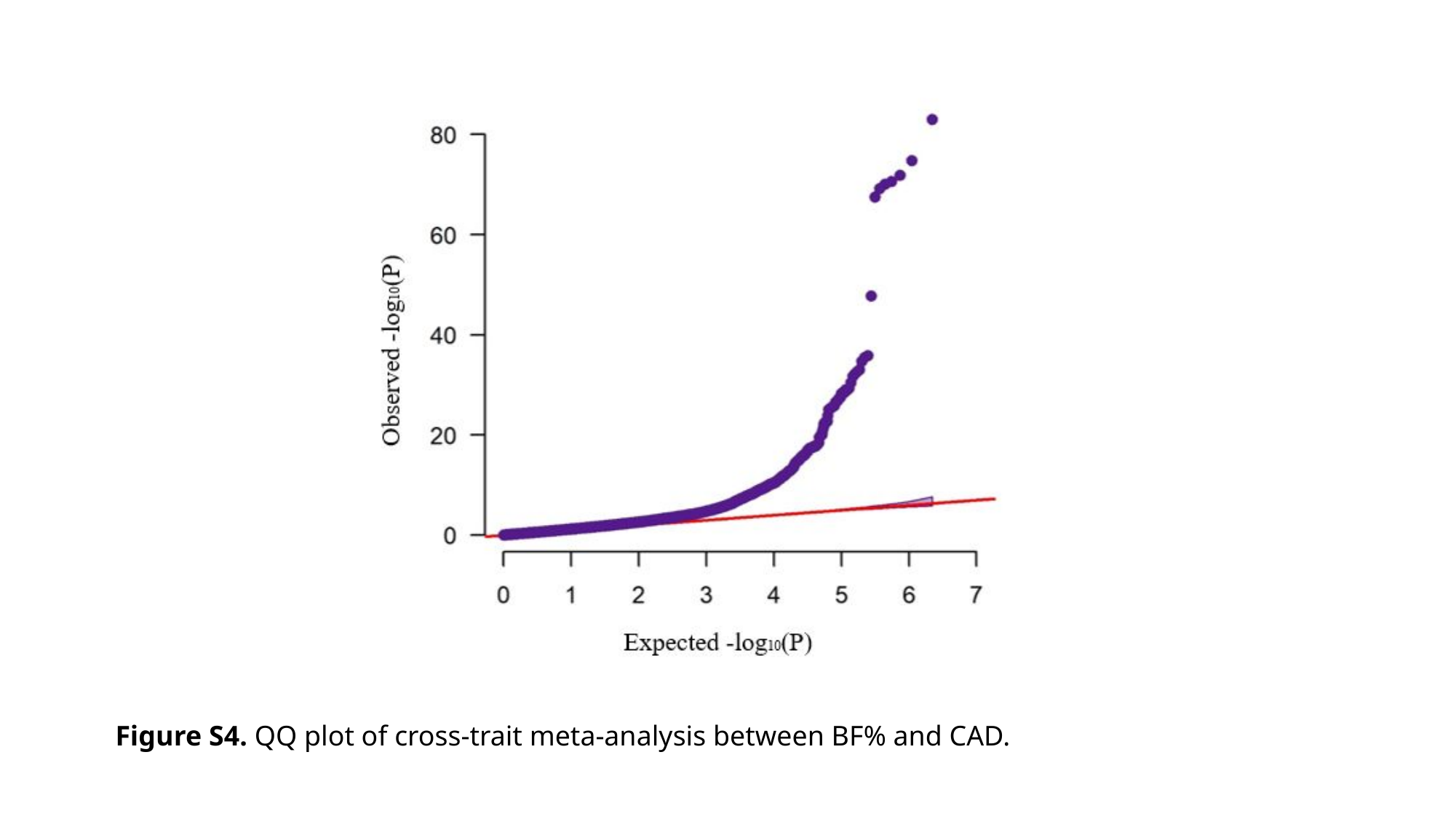

Figure S4. QQ plot of cross-trait meta-analysis between BF% and CAD.
